# Avaliação da dor após radiofrequência pulsátil nos pacientes com osteoartrite do quadril

**DOI:** 10.1055/s-0045-1809516

**Published:** 2025-06-23

**Authors:** Rafaela Reis Torrealba, Phercyles Veiga-Santos, Maria Isabella Cruz de Castro, Lourenço Peixoto, Marcelo Felipe Almeida, Conrado Torres Laett

**Affiliations:** 1 Instituto Nacional de Traumatologia e Ortopedia Jamil Haddad, Rio de Janeiro, RJ, Brasil

**Keywords:** dor crônica, osteoartrite do quadril, quadril, tratamento por radiofrequência pulsada, chronic pain, hip, osteoarthritis, hip, pulsed radiofrequency treatment

## Abstract

**Objetivo** Avaliar o papel da radiofrequência pulsátil (RFP) no manejo álgico de pacientes com osteoartrite (OA) do quadril com indicação cirúrgica.

**Métodos** Foram selecionados 30 pacientes da fila de cirurgia para artroplastia total de quadril com tempo de espera de 1 a 3 anos. O grau de OA foi mensurado radiograficamente de acordo com a classificação de Tönnis. As RFPs foram realizadas no centro cirúrgico por dois cirurgiões seniores especialistas em quadril do hospital. O procedimento foi guiado por fluoroscopia e ocorreu sob sedação anestésica. Todos os pacientes foram avaliados no pré- e pós-procedimento através do questionário Short Form-36 por uma única enfermeira.

**Resultados** Dos 30 pacientes previamente selecionados, apenas 13 realizaram a RFP. Dentre eles, um era classificado como Tönnis tipo I, quatro como II e oito como III. Os resultados evidenciaram a melhora do quadro álgico em apenas 6 pacientes (46%), do estado geral de saúde em 9 (69%), dos aspectos sociais em 8 (62%) e da saúde mental em 3 (8%). Além disso, 2 pacientes (15%) chegaram a relatar piora do quadro álgico após a RFP e 3 (23%) relataram piora do estado de saúde geral.

**Conclusão** Em graus mais avançados de degeneração articular do quadril (Tönnis III) a técnica se mostrou falha, arriscada e insatisfatória. Os dados obtidos colocam em questão não só o custo-efetividade da técnica, mas sua indicação para os pacientes com OA do quadril como tratamento conservador alternativo seguro e eficaz.

## Abstract (en)

**Objective** To evaluate the role of pulsed radiofrequency (PRF) in the pain management of patients with hip osteoarthritis (OA) and surgical indication.

**Methods** We selected 30 patients from the waiting list for total hip arthroplasty, with wait time ranging from 1 to 3 years. The OA degree was measured radiographically according to the Tönnis classification. Patients underwent PRF in the surgical center, performed by two senior hip surgeons from the hospital. The procedure was fluoroscopy-guided and occurred under anesthetic sedation. One nurse assessed all patients before and after PRF using the short form-36 questionnaire.

**Results** From the initial sample of 30 patients, only 13 underwent PRF. Per the Tönnis classification, one subject was type I, four were type II, and eight were type III. The results showed an improvement in pain in 6 patients (46%), general health status in 9 (69%), social aspects in 8 (62%), and mental health in 3 (8%). Furthermore, 2 subjects (15%) reported pain worsening after PRF, and 3 (23%) reported general health status worsening.

**Conclusion** In more advanced degrees of hip joint degeneration (Tönnis III), the technique was flawed, risky, and unsatisfactory. The data obtained question PRF cost-effectiveness and its indication for patients with hip OA as a safe and effective alternative conservative treatment.

## Introdução

Com o processo de envelhecimento populacional e o aumento das exigências físicas em atividades esportivas e laborais, a osteoartrite (OA) do quadril vem aumentando de forma considerável. É sabido que a prevalência aumenta com a idade e que, após os 85 anos, 1 em cada 4 indivíduos apresentam OA sintomática do quadril. A incidência da coxartrose apresenta grande variação entre estudos populacionais, provavelmente devido à dissociação clínico-radiológica. Como consequência, dores crônicas, rigidez, limitação do arco de movimento e instabilidade, são problemas a serem enfrentados. 1

Dor crônica no quadril representa uma prevalência estimada de 7% em homens e 10% em mulheres na população acima de 45 anos de idade. 2 A qualidade de vida desses pacientes está diretamente associada à duração do quadro álgico e à necessidade de prolongadas buscas a estratégias conservadoras para o alívio da dor, como fisioterapia, anti-inflamatórios não esteroidais, opiáceos e injeções intra-articulares de corticoide. Com frequência, esses métodos apresentam alívio parcial e muito limitado dos sintomas. 3

Diversas fontes álgicas intra- e extra-articulares são focos primários de dor no quadril, o que torna difícil diferenciá-los. Por esse motivo, a radiofrequência (RF) e as infiltrações intra-articulares são postas em prática, a fim de elucidar o foco álgico responsável. Apesar da literatura ainda ser muito controversa, a RF vem cada dia sendo mais utilizada como alternativa para tratar a dor articular, quando é refratária às outras medidas conservadoras disponíveis e nos casos em que o tratamento cirúrgico não é aconselhável. 4 5

Foi determinado através de modelos anatômicos que a anatomia sensitiva responsável pela inervação da articulação do quadril se dá através dos ramos capsulares dos nervos femoral e obturatório, sendo esses os grandes pontos alvos a serem neuromodulados na RF, guiados por imagem fluoroscópica em anteroposterior (AP) da bacia. 1

A classificação de Tönnis é uma das mais conhecidas e utilizadas em todo mundo na avaliação da OA do quadril. A contemplação de simples radiografia em incidência AP da bacia é o suficiente para sua classificação, inicialmente descrita em três graus progressivos de degeneração articular. Em 1999 foi adicionado o grau 0, correspondendo aos indivíduos sem a doença. O tipo I corresponde aos pacientes com OA leve, mínima redução do espaço articular, aumento da esclerose e ausência ou mínima perda da esfericidade da cabeça femoral. O tipo II demonstra OA moderada, com pequenos cistos, moderado estreitamento do espaço articular e moderada perda da esfericidade da cabeça. O tipo III são os pacientes com a doença em estágio avançado, o que inclui OA severa, com grandes cistos, severo estreitamento do espaço articular, severa perda da esfericidade da cabeça e necrose avascular. 6

Este estudo teve como objetivo principal avaliar a melhora da qualidade de vida dos pacientes com OA do quadril submetidos à RF pulsátil de forma imediata, em 2, 4 e em 6 meses após o procedimento, através do questionário Short-Form 36 (SF-36). De forma secundária, estabelecer um protocolo para a realização da RF no hospital e sanar relativamente a demanda álgica dos pacientes que aguardam na fila para a cirurgia.

## Materiais e Métodos

Este estudo observacional e não controlado foi realizado nas dependências do mesmo hospital entre maio e setembro de 2022. Elegemos dois cirurgiões de quadril sênior do mesmo grupo de cirurgia do quadril para conduzirem o trabalho. Uma primeira avaliação quanto à qualidade de vida de todos os pacientes foi realizada no préprocedimento, por uma única enfermeira, através do questionário SF-36. Todos os pacientes assinaram um termo de consentimento livre e esclarecido (TCLE) e o trabalho obteve aprovação do Comitê de Ética, afiliado à Plataforma Brasil, sob o parecer número 6.145.444 e CAAE n° 69626023.1.0000.5273.

### Pacientes

Foram selecionados primariamente 30 pacientes com coxartrose, de ambos os sexos, aguardando para cirurgia na fila de artroplastia total do quadril (ATQ). Os critérios de inclusão utilizados foram idade acima de 50 anos, apresentar a doença de forma primária no quadril e estarem aguardando entre 1 e 3 anos na fila cirúrgica para a ATQ. Foram excluídos os pacientes abaixo de 50 anos, com coxartrose secundária, cirurgias prévias no quadril a ser abordado, infiltração anestésica há menos de 6 meses, assim como tempo inferior a 1 ano e superior a 3 anos de espera na fila para a cirurgia.

### Classificação

Todos os quadris foram classificados quanto à Tönnis ( Tabela 1 ) por um médico residente em Ortopedia e Traumatologia do mesmo hospital, através do programa de imagem MDICON.

**Tabela 1:** Classificação de Tönnis

Classificação	Significado
0	Sem sinais de OA do quadril.
I	- OA leve. - Estreitamento mínimo do espaço articular e leve esclerose. - Ausência ou mínima perda da esfericidade da cabeça.
II	- OA moderada. - Estreitamento moderado do espaço articular com pequenos cistos. - Moderada perda da esfericidade da cabeça.
III	- OA severa. - Severo estreitamento articular com grandes cistos. - Severa deformidade da cabeça.

**Abreviação:** OA, osteoartrite. **Notas:** Adaptado de Tönnis e Heinecke 1999. 5

### A RF Pulsátil

Cada cirurgião seria responsável por realizar 15 RFs de forma aleatória. No entanto, só foi possível a realização de 13 procedimentos, devido aos resultados parciais não satisfatórios, visto que alguns pacientes evoluíram com piora da dor. A RF pulsátil conta com um gerador com amplitude de 45 V com duração de 2 vezes por segundo. O gerador modifica parâmetros em tempo real, para atingir a temperatura local desejada. Nesse método, a temperatura máxima chegou a 42° Celsius sem que tenha ocorrido dano irreversível aos tecidos nem acometimento das fibras motoras.

### O procedimento

**Fig. 1:**
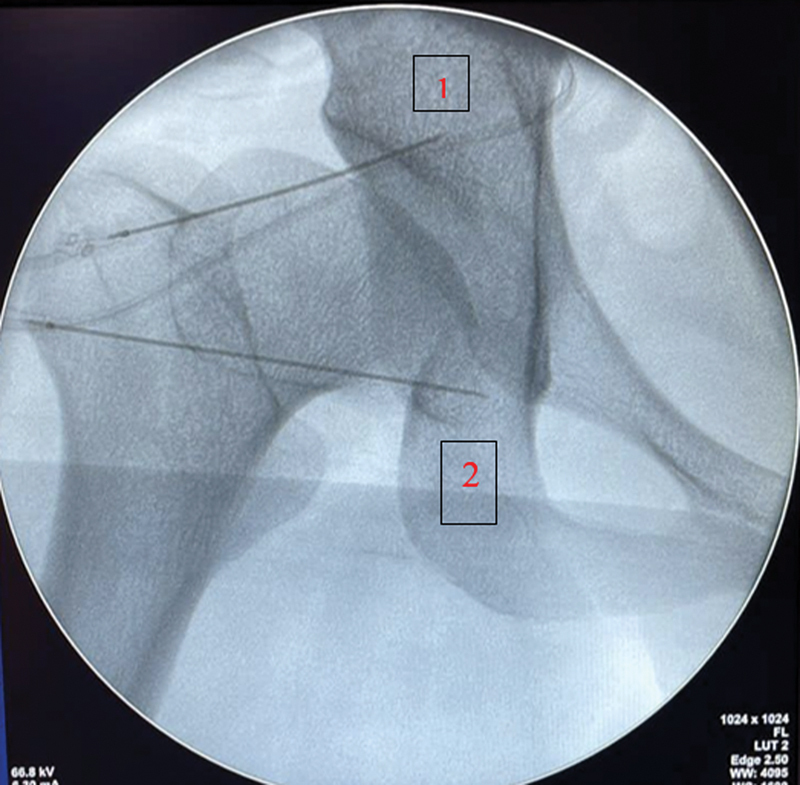
(1) Nervo femoral (2) nervo obturatório. Fonte: Imagem fluoroscópica do hospital.

Todos os pacientes foram levados ao centro cirúrgico e submetidos à leve sedação durante todo o procedimento e anestesia local com lidocaína 2% no quadril a ser infiltrado. As ponteiras de RF pulsátil utilizadas foram da marca Sollievo (Sollievo Medicina Especializada, São Caetano do Sul, SP, Brazil), sendo duas destinadas a cada indivíduo. Uma foi introduzida nos ramos sensitivos do nervo obturatório, imediatamente inferior à gota de lágrima e outra nos ramos sensitivos do nervo femoral, inferomedial à espinha ilíaca anteroinferior (EIAI). Os parâmetros anatômicos foram obtidos através de uma imagem fluoroscópica em AP do quadril ( Fig. 1 ). Após o posicionamento das ponteiras, foi preconizado o estímulo teste sensitivo com 50 Hz, a fim de provocar parestesia ou dor no quadril à 0,5 V. Em seguida, outro estímulo com 2 Hz para certificar-se que não havia nenhuma fibra motora envolvida, foi realizado. Por fim, a neuromodulação foi alcançada após 300 segundos em cada nervo com temperatura de 42°C. Priorizando evitar efeitos adversos, tais com dor por desaferentação e hematoma local, uma solução com 2 ml de metilprednisolona 20 mg e ropivacaína 1% foi injetada em cada cânula e um curativo compressivo foi realizado.

### Short Form-36

O SF-36 é uma medida da qualidade de vida amplamente utilizada, desenvolvida nos anos 80 nos EUA. O questionário conta com 11 questões e 36 itens, dos quais oito componentes (domínios ou dimensões) são representados por capacidade funcional (10 itens), aspectos físicos (4 itens), dor (2 itens), estado de saúde geral (5 itens), vitalidade (4 itens), aspectos sociais (2 itens), aspectos emocionais (3 itens), saúde mental (5 itens), além de uma questão comparativa sobre a percepção do paciente em relação a sua atual saúde e a de um ano atrás. 7 8

### Análise Estatística

Os valores de cada domínio do SF-36 são discretos e limitados ao intervalo entre 0 e 100. Além disso, os dados apresentam distribuição não-normal de acordo com o teste de Shapiro-Wilk. Assim, os valores serão reportados como mediana (intervalo interquartílico [IIQ]), a comparação entre os valores pré- e pós-intervenção será feita através do teste de postos pareados de Wilcoxon e a associação entre as mudanças em cada domínio, através do coeficiente de correlação de postos de Spearman.

## Resultados

Nossos resultados elucidam apenas 13 pacientes dos 30 previamente selecionados. Dentre eles, oito apresentaram classificação de Tönnis tipo III, quatro classificação II e um paciente apresentou classificação tipo I. Todas as avaliações do pré-SF-36 foram feitas no dia ou no dia anterior ao procedimento, enquanto para as avaliações pós-procedimento foi utilizado um intervalo mediano de 49 dias, sendo o menor e o maior intervalo, respectivamente, de 25 e 96 dias.

**Fig. 2:**
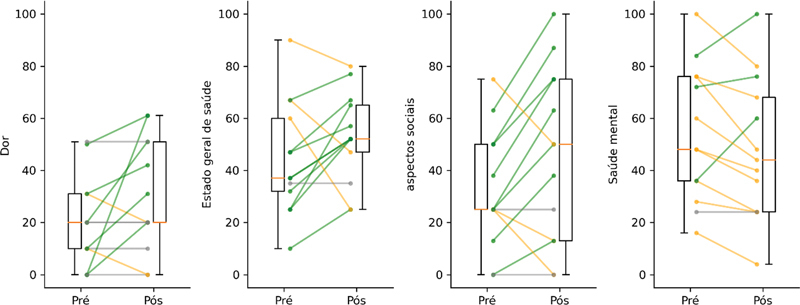
Variação nos domínios avaliados nos momentos pré e pós-cirúrgicos. Os valores do grupo estão apresentados por diagramas de caixa ( *boxplot* ) e os valores individuais através das linhas. As linhas verdes indicam melhora no domínio, as laranjas indicam piora e as cinzas indicam manutenção dos valores.

Os valores do domínio dor e outros domínios analisados podem ser vistos na Tabela 2 . Observamos melhora no quadro álgico em apenas 6 pacientes (46%), manutenção do estado pré-cirúrgico em 5 (38%) e piora em 2 (15%). O estado geral de saúde melhorou em 9 pacientes (69%), se manteve idêntico em 1 (8%) e piorou em 3 (23%). Os aspectos sociais melhoraram em 8 pacientes (62%), se mantiveram idênticos em 2 (15%) e pioraram nos outros 3 (23%). A saúde mental, por sua vez, apresentou melhora em apenas 3 pacientes (23%), manutenção em 1 (8%) e piora nos demais 9 (69%). A variação individual nos pacientes pode ser vista na Fig. 2 .

**Tabela 2:** Domínios da qualidade de vida

	Pré	Pós	Valor de *p*
**Dor**	20 (21)	20 (31)	0,057
**Estado geral de saúde**	37 (28)	52 (18)	0,168
**Aspectos sociais**	25 (25)	50 (62)	0,053
**Saúde mental**	48 (40)	44 (44)	0,169

**Abreviação:** p, valor p do teste de postos pareados de Wilcoxon.

**Fig. 3:**
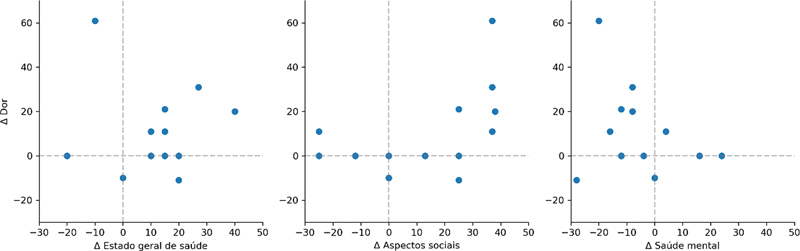
Diagrama de dispersão das variações nos domínios avaliados. Cada ponto indica um indivíduo e as linhas pontilhadas de referência indicam a manutenção dos valores entre o pré e pós cirúrgicos. Pontos situados no quadrante superior direito indicam indivíduos que apresentaram melhora simultânea nos dois domínios, enquanto pontos situados no quadrante inferior esquerdo indicam piora simultânea.

Não observamos correlação entre a variação no domínio dor e as variações nos domínios estado geral de saúde e saúde mental ( Fig. 3 ). Observamos um valor limítrofe no teste estatístico de correlação entre as variações no domínio dor e aspectos sociais ( *p* = 0,056).

O estudo foi interrompido devido à ausência de resultados parciais benéficos ao paciente após 43,3% das RFs realizadas.

## Discussão

De acordo com Giaccari et al., 10 é sabido mundialmente que a osteoartrite (OA) é a desordem articular mais prevalente no mundo e uma das principais causas de morbidade e incapacidade funcional. A coxartrose a segunda forma mais comum de OA. Em 2019, a prevalência de osteoartrite dos últimos 10 anos, aumentou 113,25%, passando de 247,51 milhões de afetados em 1990, para 527,81 milhões em 2019. Dessa forma, a documentação literária dos gastos nos serviços de saúde ao que norteia o tema, representa 1 a 2,5% do produto nacional bruto dos países desenvolvidos, esperando-se quadruplicar esse valor até 2030. 11

O tempo de espera para a ATQ em nosso hospital, desde o momento da inserção na fila até a cirurgia, nos últimos 10 anos, é de 3,1 anos. Com frequência, esses pacientes com dor crônica, são levados ao uso excessivo de medicações analgésicas e anti-inflamatórias, além de sucessivas infiltrações intra-articulares com corticosteroides, as quais apresentam ação restrita. Levando em consideração essa realidade atual em nosso hospital, vislumbramos a radiofrequência pulsátil (RFP) como possível método alternativo e eficaz, para a melhora álgica desses pacientes e, consequentemente, da qualidade de vida.

Apesar de a RF ser uma técnica comumente usada para dor musculoesquelética crônica, 10 pouco se é encontrado na literatura quanto ao tratamento com a RFP nos pacientes com coxartrose. Em 2017, Short et al. 1 e Bhatia et al. 3 revisaram 14 artigos e demostraram o grande potencial na redução álgica secundária à osteoartrite em até 3 anos, além da melhora na deambulação, realizando-se a RF na inervação sensorial do quadril (nervos obturatório, obturatório acessório e femoral). Complicações do procedimento são raras e envolvem lesão vascular, neurite, formação de hematoma e ablação inadvertida de ramos motores dos nervos obturatório e femoral.

A grande vantagem da RFP consiste nos efeitos de neuromodulação do campo elétrico local por alteração da transmissão sináptica e, consequentemente, menos danos aos tecidos locais e menos dor por desaferentação. 12 A RF resfriada (RFR) é semelhante à pulsátil, porém refrigerada à água através de uma sonda, podendo atingir 60°C. Também possibilita gerar uma lesão neuronal de maior área, mas seu custo pode chegar a quase o dobro do valor da pulsátil. 13

Em outro estudo publicado em 2015, 15 pacientes com OA do quadril leve a moderada, tipos I e II de Tönnis, foram submetidos à RF pulsátil (RFP) e comparados com 14 não submetidos ao procedimento, tratados conservadoramente com paracetamol, anti-inflamatórios não esteroidais (AINEs) e opioides. Os pacientes foram avaliados quanto ao nível de dor e função do quadril através da escala analógica visual (EVA) e escore de Oxford (OHS) e medicamentos para dor foram utilizados no pré-procedimento e em 1, 4 e 12 semanas após. Foi observada uma melhora significativa da dor e da função do quadril quanto à EVA e à OHS nos pacientes submetidos à RFP em todas as semanas avaliadas. Esses pacientes também utilizaram menos analgésicos após o procedimento. 14

Foram constatadas algumas limitações no presente estudo. É sabido, que as ponteiras de RF resfriadas, conseguem atingir diâmetros de neuromodulação maiores do que as de RFP. Porém, devido ao alto custo, não pode ser utilizada. Como em nosso trabalho 61,5% dos quadris eram classificados como Tönnis tipo III, gravemente degenerados, acreditamos fortemente que a técnica em questão não tenha função nesses casos. Talvez seja de grande valia novos estudos em grupos com quadris menos degenerados, a fim de validar esse procedimento como tratamento alternativo para a coxartrose.

## Conclusão

O presente trabalho necessitou ser interrompido com apenas 43,3% dos pacientes programados para a realização da RF, devido aos resultados parciais desencorajadores. Portanto, deve-se questionar se o investimento nessa técnica, considerada de altíssimo custo, é de grande valia e efetivo na melhora da qualidade de vida dos pacientes com osteoartrite do quadril.
